# Factors affecting unmet need for family planning in Eastern Sudan

**DOI:** 10.1186/1471-2458-13-102

**Published:** 2013-02-04

**Authors:** Abdel Aziem A Ali, Amira Okud

**Affiliations:** 1 Faculty of Medicine, Kassala University, Kassala, Sudan; 2 Department of Obstetrics and Gynecology, Faculty of Medicine, Kassala University, P.O. Box 496m, Kassala, Sudan

**Keywords:** Unmet need, Family planning, Contraception, Sudan

## Abstract

**Background:**

In the developing countries millions of women in the reproductive age who don’t use contraceptives prefer to postpone or limit their birth. This indicates their failure to take necessary decision to prevent and avoid unwanted pregnancy.

**Methods:**

A community-based cross sectional household survey was conducted to investigate unmet need for family planning and associated factors and total demand for family planning in Kassala, Eastern Sudan between 1^st^ May and 31^st^ July 2012.

**Results:**

A total of 812 married women were enrolled in this study. Their mean age and parity was 31.8 (7.3) and 3.4 (1.8) respectively. Ever use of contraception was 25.4% (206/812) and 26.2% (213/812) were currently using contraception. Unmet need for spacing was 15.1% while unmet need for limiting was 0.7%. The pregnant and amenorrheic women whose the pregnancy or birth was unwanted and mistimed were 105 (13%) and 130 (16%) respectively. Using Westoff model the total unmet need was estimated as 44.8%. The total demand for family planning was 71%.

In logistic regression model, while age, age at marriage, parity, residence and experience of child death were not associated with total unmet need for family planning, women education < secondary level (OR=7.8; CI=5.6-10.9; *P*=0.00), husband education < secondary level (OR=1.9; CI=1.3-2.6, *P* = 0.00) and woman’s occupation; housewife (OR=4.3; CI=2.5-7.2; *P*=0.00) were associated with the total unmet need.

**Conclusions:**

Unmet need for family planning in Eastern Sudan was significantly higher among women with less than secondary education. Also; it is influenced by couple’s educational status and woman’s occupation. The results of this study necessitate the need for the programme managers to take into account the concept of reproductive health education.

## Background

In the developing countries millions of women in the reproductive age who don’t use contraceptives prefer to postpone or limit their birth. This indicates their failure to take necessary decision to prevent and avoid unwanted pregnancy [1]. Unmet need for family planning is defined as percentage of all fecund women who are married or living in union and thus presumed to be sexually active but are not using any method of contraception, either do not want to have more children or want to postpone their next birth for at least two more years or do not know when or if they want another child [2]. One of the sequels of unmet need is unwanted pregnancy with its serious consequences [3]. Globally 50 million women resort to induced abortion and ultimately results in high maternal morbidity and mortality [4,5]. Thus, family planning and spacing among births are one of the methods to avoid these deaths. In Sudan maternal mortality and fertility indicators are among the highest in the world. The Sudan household survey in 2010 showed only 9% of women aged 15-49 year used a contraceptive method and the unmet need for contraception (either spacing or limiting births) is 29% and the total fertility rate is 5.6 per children woman [6]. Eastern Sudan is an area of high maternal mortality ratio (713 per 100.000 live births) and low use of contraception (44%) [7,8]. Family planning services were introduced in Sudan in 1965 with the foundation of the Sudan Family Planning Association, which provides services throughout the country. Despite the great effort of the government to distribute knowledge and information of the family planning through the different media there is no improvement in the use of contraception and the availability and\or accessibility is still vary in particular between the rural and urban areas [9]. To our knowledge no previous study has documented the factors affecting unmet need for family planning in Sudan; thus this survey is expected to provide the stakeholders and programme managers with fundamental data necessary for intervention to improve the reproductive health in Eastern Sudan.

## Methods

### Study design

A community-based cross sectional household survey was conducted to investigate unmet need for family planning, factors affecting unmet need and total demand for family planning in Kassala State, Eastern Sudan between 1^st^ May and 31^st^ July 2012. Random selection of three villages with different distance (not less than 10 km) from Kassala, the capital city of the State and two towns including Kassala itself was used.

### Setting

Kassala is 600 killometer from Khartoum on Ethiopian –Eritrean border with 1.8 million inhabitants, 440491 out of them are women in the reproductive age [10]. In Eastern Sudan there is a prominent diversity in culture, religion, language and ethnicity and some tribes are nomads. The maternal mortality is one of the highest in country. In Kassala there are 28 health centers and more than ten hospitals providing health services and there is an office of Sudanese family planning association providing different aspect of services like pills and intrauterine contraceptive device free of charge but the most recent methods such as implant and marina are not available. The family planning services are offered by different health personnel (doctors, nurses, midwives). After obtaining verbal consent structured questionnaires were used to gather data from all ever married women of childbearing age (15-49 years) to identify unmet need for family planning and associated factors (age, parity, education and husband education). Irrespective of its validity the age of the women defined as age completed in year at the time of interview using the recall method.

### Concept of unmet need for family planning

Unmet need for family planning was defined as the percentage of all fecund married women who are not using an appropriate method of contraception even though they do not want to get pregnant also it included the pregnant and amenorrheic women whose the pregnancy or birth was unwanted or mistimed. Women who are not using an appropriate method of contraception and wanted to wait for at least two years or who wanted no more children were subcategorized as unmet need for spacing and limiting respectively. In this study the concept of unmet need was applied to ever married women in the union and it was estimated using Westoff model [11]. Total demand for family planning was calculated as sum of the percent of unmet need plus percent using contraception.

The surveyed women were first divided into those using a contraceptive method and those not using a method. The nonusers were then subdivided into pregnant or amenorrheic women and nonusers who were neither pregnant nor amenorrheic category at the time of the survey. The pregnant or amenorrheic were further subdivided in to three categories: those their pregnancy was intended, mistimed and unwanted at the time of the survey. Those in the mistimed and unwanted pregnancy category were regarded as one component of the total unmet need. The other component consists of nonusers who were neither pregnant nor amenorrheic. These women were first divided into fecund or infecund women, with the fecund women then subdivided by their reproductive preference. Those who wanted another child soon were excluded from the unmet need category, while women who wanted to wait for at least two years or who wanted no more children were classified in unmet need group.

### Statistical analysis

Data were entered into a computer database and SPSS software (SPSS Inc., Chicago, IL, USA, version 13.0, free version one) and double checked before analysis. Unmet need was the dependent variable and socio-demographic characteristics were independent variables. Confidence intervals of 95% were calculated and *P*<0.05 was considered significant. In case of discrepancy between the results of ANOVA and x^2^ test and the results of multivariate analyses, the later was taken as final. Means and proportions for the socio-demographic characteristics were compared between the unmet need for spacing and met-need catogery using student and x^2^ test, respectively and *P*<0.05 was considered significant.

### Ethics

The study received ethical clearance from the Research Board at Ministry of Health Kassala State, Eastern Sudan.

## Results

**Figure 1:**
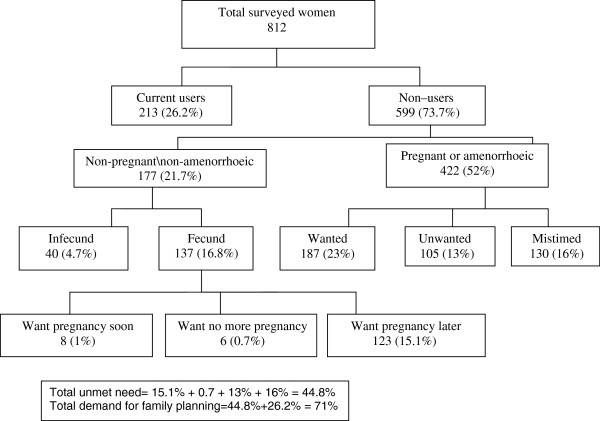
Unmet need among ever married woman in Eastern Sudan, 2012 (Westoff model).

A total of 812 married women were enrolled in this study. Their mean age and parity was 31.8 (7.3) and 3.4 (1.8) respectively. The majority were of secondary education (418/812, 51.5%), of urban residence (496/812, 61.1%) and housewives (730/812, 89.9%). Ever use of contraception was 25.4% (206/812) and 26.2% (213/812) were currently using contraception. Using Westoff model the total unmet need was estimated as 44.8%, Figure 1. Unmet need for spacing was 15.1% while unmet need for limiting was 0.7%. The total demand for family planning was 71%.

In logistic regression model, while age, age at marriage, parity, residence and experience of child death were not associated with total unmet need for family planning, women education < secondary level (OR=7.8; CI=5.6-10.9; *P*=0.00), husband education < secondary level (OR=1.9; CI=1.3-2.6, *P* = 0.00) and woman’s occupation; housewife (OR=4.3; CI=2.5-7.2; *P*=0.00) were associated with the total unmet need, Table 1.

**Table 1:** Factors affecting total unmet need for family planning in Eastern Sudan using univariate and multivariate analyses, 2012

**Variable**	**Univariate analyses**				**Multivariate analyses**						
	**OR**	**95% CI**	***P*-value**	**OR**	**95% CI**	***P*-value**					
Age, years	1.00	.98-1.02	0.679	1.01	.99-1.04	0.134
Age at marriage, years	1.04	.98-1.03	0.570	1.01	.98-1.04	0.311
Parity ≥3	.701	.52-.92	0.014	0.92	.66-1.30	0.665
Women's education < secondary level	8.10	5.91-11.11	0.000	7.86	5.62-10.98	0.000
Husband’s education< secondary level	2.00	1.51-2.65	0.000	1.91	1.36-2.67	0.000
Rural residence	.993	.87-1.12	0.909	1.00	.87-1.14	0.972
Occupation, housewife	3.39	2.12-5.44	0.000	4.32	2.58-7.23	0.000
Experience of child death	.893	.35-2.24	0.809	1.03	.35-2.98	0.954

Abbreviations: OR, Odds Ratio; CI, confidence interval.

Both woman and husband educations < secondary level and age at marriage were significantly associated with unmet need for spacing (*P*=0.00, 0.006 and 0.03 respectively) however age, parity, residence, occupation and experience of child death showed no significant statistical difference between the group of met need and unmet need for spacing, Table 2.

**Table 2:** Factors affecting unmet need for spacing in Eastern Sudan, 2012

**Variable**	**Unmet need for spacing *(N*=123)**	**Met need (*N*=448)**	***P***
Age, years	32.3 (7.9)	31.9 (7.3)	0.6
Age at marriage, years	23.1 (6)	21.9 (5.4)	0.03
Parity	3.2 (1.5)	3.5 (1.9)	0.08
Rural residence	61 (49.5%)	173 (38.6%)	0.05
Mother education, < secondary	105 (85.5%)	121 (27%)	0.00
Husband education, < secondary	67 (54.5%)	185 (41.2%)	0.006
Occupation, housewife	113 (91.9%)	376 (83.9%)	0.08
Experience of child death	3 (2.4%)	11 (2.4%)	0.6

· Data was shown in mean (SD) and number (%).

## Discussion

The current study revealed that 44.8% of ever married woman in reproductive age in Eastern Sudan at time of survey had unmet need for family planning. The total unmet need for family planning in this study is greater than the regional (30.7%) and national (35%) in 2005 and 2011 respectively [12,13]. In West Africa, unmet need ranged from 16% to 34% while in Southern Africa it ranged from 13% to 38% [11]. Thus policy-makers and programme managers should carefully consider the unmet need for family planning of target populations when making decisions about service integration. Yet, it might be difficult to integrate and expand family planning services into Eastern Sudan and make the result of unmet need comparable with African countries because Sudan has one of the lowest net school enrollment rates for girls in the world [14] and this will ultimately shift the girls away from education, the accurate factor affect the woman's ability to make her own decision regarding the reproductive health. Higher proportion was observed for unmet need for spacing in comparison with that for limiting (15.1% Vs 0.7%). This observation is in agreement with other reports from different low-income countries, such as neighboring Ethiopia where unmet need for spacing contributed for two third of the total unmet need [15]. The total demand for family planning in the current study is 71% which is higher than the 49.6% reported among Ethiopian women [15]. The discrepancy appeared because of the high percentage of unmet need for family planning in Eastern Sudan. This difference may be due to regional, cultural variation between the two neighboring areas or efforts exerted by the regional Reproductive Health Department in Ministry of Health, Eastern Sudan.

In Eastern Sudan couples education was the main predictor of the low use of family planning [8]. Husband non approval and religious belief were influential determinants for family planning [8]. Husbands and women educational status and woman’s occupation were found to influence unmet need for family planning. In most population the unmet need declined with increasing woman’s education; for instance in Uganda it was lower among women with secondary or higher education and in Kenya the women with less than secondary education were 2 times more likely to experience unmet need for family planning in comparison with those with secondary level [16,17]. The educated women have better access to health facilities and information about the contraception. Likewise husband’s education in many African countries as well as in the current study was associated with utilization of family planning [18]. Unfortunately there is high level of illiteracy among Sudanese women and illiteracy was the significant predictor for maternal morbidity and mortality in the different regions of Sudan including the eastern part [19,20]. Sudan has one of the lowest net school enrollment rates for girls in the world and this might be due to early marriage and poverty therefore consideration of the education in health programmes is an urgent need and priority. Most of the socio-demographic factors such as woman’s occupation are relatively affecting the reproductive health and women’s decision-making power [21]. This power can inhibit the ability of the women to seek health services and thus contributes to low use of contraception and high percent of unmet need for family planning.

This study has some limitations that might weaken the results. The men were not included as participants to understand their perception towards the total unmet need for family planning. The overlapping between some urban and rural areas or even the nomads might affect our results. Also the study did not include the unmarried women because of the fact that the community and the religion reject the sexual activities outside marriage. Lastly the unmet need for limiting was not compared with the total met need for family planning because of its small proportion.

## Conclusions

Despite the majority of the respondents have secondary education; unmet need for family planning in Eastern Sudan was significantly higher among women with less than secondary education. Also; it is influenced by couple’s educational status and woman’s occupation. The results of this study necessitate the need for the programme managers to take into account the concept of reproductive health education.

## Competing interest

We declare that we have no conflict of interest.

## Authors’ contributions

AAA carried out the study, participated in the analysis and manuscript drafting. OA participated in analysis and manuscript drafting. Both authors read and approved the final manuscript.

## Pre-publication history

The pre-publication history for this paper can be accessed here:

http://www.biomedcentral.com/1471-2458/13/102/prepub
